# CAR-T cell therapy in relapsed or refractory multiple myeloma and access in Turkey

**DOI:** 10.3389/fmed.2024.1413825

**Published:** 2024-08-28

**Authors:** Goker Hakan, Kelkitli Engin, Karakulak Aladag Elifcan, Demiroglu Haluk, Turgut Mehmet, Kambhampati Suman, Krem Maxwell

**Affiliations:** ^1^Department of Hematology, Medical Faculty of Hacettepe University, Ankara, Türkiye; ^2^Department of Hematology, Medical Faculty of Ondokuz Mayis University, Samsun, Türkiye; ^3^Research Medical Center, HCA Midwest Health, Kansas City, MO, United States

**Keywords:** B-cell maturation antigen, CAR-T cell, multiple myeloma, relapse, therapy

## Abstract

The past decade has seen the development of immunotherapy for the treatment of multiple myeloma (MM), beginning with monoclonal antibodies (mAbs) in the relapsed and refractory setting and culminating in the market approval of chimeric antigen receptor T cells (CAR-T) and bispecific antibodies (BsAbs). The medical community is evaluating the efficacy and safety of these targeted immunotherapies, most of which currently target B-cell maturation antigen (BCMA) on the surface of plasma cells. Two anti-BCMA CAR-T products are available for treating relapsed or refractory MM: idecabtagene vicleucel (ide-cel) and ciltacabtagene autoleucel (cilta-cel). Ide-cel and cilta-cel demonstrate the ability to induce deep responses in heavily pretreated diseases, including patients with triple-class-refractory and penta-refractory diseases. However, there are key similarities and differences regarding these agents, unknowns regarding their comparative efficacy and toxicity, and mechanisms underlying resistance to these new immunotherapies. This review discusses CAR-T cell therapy in relapsed refractory MM, with a focus on efficacy, toxicities, and the evolving trajectories of these therapies in the USA, as well as access in Turkey.

## 1 Introduction

MM is a neoplasm that is associated with the clonal proliferation of malignant plasma cells in the bone marrow and/or extramedullary tissues. It accounts for approximately 15% of hematologic malignancies (1).

MM treatment has made great strides over the past several decades, however, despite substantial. Advanced MM remains a largely incurable disease, which underscores the unmet need for more effective treatment approaches. In recent years, T cell redirection therapies for relapsed/refractory MM (RRMM), especially CAR-T and BsAbs, have been significant advances. CAR-T cell therapy involves the modification of patient or donor T cells to target specific cell surface antigens (2, 3) and our review will focus on the two approved products in the USA.

Since 2021, CAR-T has emerged as a promising immunotherapy for RRMM. Currently approved products are autologous, where T cells obtained from patients are genetically manipulated to a specific tumor-targeted receptor called the CAR (4).

In 2021 and 2022, the Food and Drug Administration (FDA) authorized two CAR-T products: ide-cel and cilta-cel in RRMM, both of which are directed against surface BCMA (4). BCMA is present in healthy and neoplastic plasma cells. It is a type III transmembrane glycoprotein member of the tumor necrosis factor receptor superfamily (TNFRSF) and is presently the target for novel T cell-directed therapies (RRMM) (5). Anti-BCMA CAR-T cell therapy has obtained significant responses in RRMM (6).

## 2 Overview of pivotal trials leading to approval of BCMA CAR-T

### 2.1 Ide-cel

Ide-cel is a BCMA CAR with a 4-1BB costimulatory domain (7). On 26 March 2021, Ide-cel, the first CAR-T product FDA-approved for RRMM, was approved for patients who had four or more prior lines of therapy, based on a phase 2 study involving patients who had received at least three prior regimens, based on the KarMMa-1 data (8).

KarMMa-1 was a Phase I/II global trial, where patients were eligible if they had three or more prior lines of therapy and were previously exposed to an IMID, proteasome inhibitor (PI), and anti-CD28 mAb. There were 128 enrolled patients who received ide-cel at three dose levels (target doses of 150 × 10^6^ to 450 × 10^6^) with a median follow-up of 13.3 months. The overall response rate (ORR) was 73% with 33% complete response (CR) or better. The minimal residual disease (MRD)-negative status defined according to IMWG criteria (9) was confirmed for 33 patients and was 25% for the cohort and 79% of those in CR, indicating deep responses.

The progression-free survival (PFS) for all dose levels was 8.8 months (95% confidence interval: 5.6–11.6). The most common adverse effects among the 128 treated patients included neutropenia in 117 patients (91%), anemia in 89 (70%), and thrombocytopenia in 81 (63%). Cytokine release syndrome (CRS) was reported in 107 patients (84%), including 7 (5%) who had events of grade 3 or higher. Neurotoxic effects developed in 23 patients (18%) and were of grade 3 in 4 patients (3%); no neurotoxic effects higher than grade 3 occurred (4).

A higher dose of 450 × 106 CAR+ T cells was associated with a higher ORR of 81%, a CR rate of 39%, and a longer median PFS of 12.2 months. The KarMMa-1 trial excluded patients with major comorbidities, aggressive illness, or a history of BCMA exposure. Real-world outcomes of patients who did not meet the KarMMa-1 eligibility criteria and were treated with standard of care (SOC) ide-cel found similar efficacy and comparable toxicity to the trial (10). However, a study by Hashimi et al. showed that patients were more likely to advance within 3 months if they had a history of extramedullary illness, were of Hispanic ethnicity, had received bridging treatment or prior BCMA-targeted therapy, had high ferritin in lymphodepletion, had plasma cell leukemia, or t (4; 14). The median PFS was considerably lower (3.2 months vs. 14.1 months) when three or more of these variables were present. This information may be used to pinpoint individuals who are more likely to experience an early progression and provide targeted treatments to enhance results (11).

In recent years, trials with ide-cel have focused on earlier lines. In May 2024, in the USA, CAR-T is racing to earlier lines. Ide-cel is approved in the second line based on the KarMMa - 3 study (12).

Rodriguez-Otero et al. reported the KarMMa-3 outcomes from the open-label, phase 3 global study. In this patient population, ide-cel therapy resulted in a considerably longer PFS and a 51% reduction in the likelihood of disease progression or death when compared to conventional regimens. When compared to the standard regimen, the ide-cel treatment significantly improved the responses of the patients; on the previous regimen, the median relapse was 7 months, and 65% of patients experienced triple refractory disease. The KarMMa-3 study found that ide-cel therapy significantly improved PFS and response, with these benefits achieved through a single infusion (13). The safety profile of ide-cel was consistent with what was found in previous research (4, 14).

### 2.2 Cilta-cel

The cilta-cel CAR structure differs from ide-cel’s in that it has two BCMA-binding domains instead of one binding domain, although it uses a similar 4-1BB costimulatory domain (15). Cilta-cel was approved in 2022 for RRMM who had four or more prior lines of therapy. This was based on the outcomes of the CARTITUDE-1 trial, an open-label, single-arm phase 1b/2 study. Eligibility criteria were at least three prior lines of therapy, being double refractory to PI and IMID and previously exposed to IMID, PI, and CD38 mAb therapy patients, safety, and validation of the suggested phase 2 dosage (phase 1b).

Stringent CRs (sCRs) accounted for 82.5% of patients at a median follow-up of 27.7 months, with an ORR of 97.9% (15, 16). MRD-negative CR was achieved in 44.3% of patients. The median PFS was 34.9 months. OS was 70% at 27 months, but the median OS was unavailable. PFS and OS were shorter in patients with International Staging System stage III, high-risk cytogenetic, EMD, and high tumor burden (17).

CRS (grades 3–5) occurred in the majority (95%) of patients, and neurotoxicity occurred in 21% (grades 3–5 in 10%). However, neurotoxicity related to cilta-cel includes atypical neurotoxicity movement disorders, which occurred in 12.4% of cases, and signs in 12.4% of cases, in addition to the conventional immune effector cell-associated neurotoxicity syndrome (ICANS) in 16.5% of cases. Symptoms including ataxia, peripheral sensorimotor neuropathy, cranial nerve (CN) palsies (most commonly CN VII), parkinsonism, and other atypical neurotoxic findings were observed in 4% of cases; the median recovery time was 70 days (range, 2–159 days) and the median onset time was 27 days (range, 11–108) (16, 17).

Hansen et al. presented real-world data on cilta-cel. More patients had high-risk cytogenetics (41%) and extramedullary disease (EMD, 35%) compared to the CARTITUDE-1 study. A total of 55% of patients did not meet the eligibility criteria for CARTITUDE-1. Common reasons for ineligibility were reported as cytopenias (19%), prior BCMA treatment (14%), oligosecretory disease (13%), organ dysfunction (12%), and plasma cell leukemia (8%). A total of 83% of patients were treated with bridging chemotherapy (overall response rate, ORR: 28%). Lymphodepletion included bendamustine: 11%, cladribine + Cy: 4%, Cy: 4%, and fludarabine (Flu) + cyclophosphamide (Cy): 81%. The ORR was 40% CR; 62% ≥ very good PR, and ≥ 80% PR. This study demonstrated that real-world outcomes were similar to those of the clinical trial, despite a higher prevalence of high-risk characteristics than trial participants. Patients who received FluCy-conditioned items had greater response rates (89%) (18). With the efficacy outcomes in CARTITUDE-1, there was a need to study cilta-cel in earlier trials.

San-Miguel et al. (19) reported the CARTITUDE-4 outcomes of phase 3, a randomized, open-label trial, to compare cilta-cel with the doctor’s choice of one of two SOC regimens: daratumumab-pomalidomide-dexamethasone or pomalidomide-bortezomib-dexamethasone in patients with lenalidomide-refractory MM after one to three lines of therapy. A total of 419 participants were randomly assigned to receive either SOC or cilta-cel. A median follow-up of 15.9 months (range, 0.1–27.3) revealed the median in patients with lenalidomide-refractory MM after one to three lines of therapy. A total of 419 participants were randomly assigned to receive either normal treatment or cilta-cel. A median follow-up of 15.9 months (range, 0.1–27.3) revealed that the median PFS was 11.8 months in the SOC treatment group and not attained in the cilta-cel group. At 12 months, the PFS rates were 48.6% (95% confidence interval, 41.5–55.3) in the SOC group and 75.9% (95% confidence interval, 69.4–81.1) in the cilta-cel group (Table 1).

**Table 1:** Results of randomized KarMMa-3 and CARTITUDE-4 studies (20).

	CARTITUDE-4		KarMMa-3	
	Cilta-Cel	SOC	Ide-Cel	SOC
Inclusion criteria	2–4 prior lines including PI + IMiD + Dara		1–3 prior lines, Len-refractory	
Therapy	CAR-T	DPd or PVd	CAR-T	DPd, DVd, IRd, Kd, or EPd
CAR-T dose, median (range)	0.71×10^6^/kg	N/A	445×10^6^(175−529× 10^6^)	N/A
Lymphodepleting chemotherapy	Flu 30 mg/m2 + Cy 300 mg/m2 for 3 d		Flu 30 mg/m2 + Cy 300 mg/m2 for 3 d	
ORR, *n* (%)	176 (85)	142 (67)	181 (71)	55 (42)
CR, *n* (%)	152 (73)	46 (22)	98 (39)	7 (5)
VGPR, *n* (%)	169 (81)	96 (46)	153 (60)	20 (16)
MRD-negative 10^5^, *n* (%)	126/144 (88)	33/101 (33)	51/254 (20)	1 (1)
Progressive disease as best response, n (%)	17 (8)	6 (3)	24 (9)	10 (8)
DOR, mo, median	Not reached; 85% at 12 mo	Not reached; 63% at 12 mo	14.8	9.7
PFS, mo, median	Not reached; 76% at 12 mo	11.8 49% at 12 mo	13.3	4.4
OS, mo, median	Not reached; 84% at 12 mo	Not reached; 84% at 12 mo	NR	NR

N/A indicates not applicable; NR, not reported; Flu, fludarabine; Cy, cyclophosphamide; DPd, daratumumab, pomalidomide, dexamethasone; DVd, daratumumab + bortezomib + dexamethasone; IRd, ixazomib + lenalidomide + dexamethasone; Kd, carfilzomib + dexamethasone; EPd, elotuzumab + pomalidomide + dexamethasone; PVd, pomalidomide + bortezomib + dexamethasone.

The safety signals were similar to CARTITUDE-1. Within the as-treated population, 176 patients received cilta-cel; 16 (9.1%) developed CN palsy (grade 2, 8.0%; grade 3, 1.1%); 134 (76.1%) developed CRS (grade 3 or 4, 1.1%; no grade 5); 8 (4.5%) developed ICANS (all grade 1 or 2); 5 (2.8%) developed peripheral neuropathy related to CAR-T (grade 1 or 2, 2.3%; grade 3, 0.6%), and 1 experienced movement and neurocognitive symptoms (grade 1). Based on the results of CARTITUDE-4, the FDA-approved cilta-cel in May 2024 for patients who had at least 1 prior line of therapy and were lenalidomide refractory (21). Table 2 shows the FDA-approved CAR-T cells ide-cel and cilta-cel in relapsed refractory MM.

**Table 2:** FDA-approved CAR-T cells ide-cel and cilta-cel in relapsed refractory multiple myeloma.

Study	*N*	ORR (%)	>CR (%)	Med PFS (months)	Grade > 3 CRS (%)	Grade > 3 neuro (%)	Delayed NT/Parkinson (any/>3) (%)	Grade > 5 AE (%)	Med F/U (months)	References
KARMMa	128	73	33	8.8	5	3	0	2	15.4	(4)
Ide-cel RWD	159	82	40	8.9	3	6	0	5	6.1	(18)
CARTITUDE-1	97	97	67	34.9	4	2	12/8	6	27.7	(15)
Cilta-cel RWD	177	80	40	n/a	7	8	9/1.4	9	2.3	()

N/A, not applicable; Med F/U, median follow-up; CRS, cytokine release syndrome, NT, neurotoxicity; RWD, real-world data.

### 2.3 Comparison of ide-cel and cilta-cel

Ide-cel and cilta-cel are similar antigen treatments for RRMM patients who have received many lines of therapy. They vary greatly in terms of CAR-T cell line, cell dose, and patient groups in clinical trials. KarMMa has a higher total disease stage, fewer penta-refractory people, a higher incidence of EMD, and a higher number of high-risk cytogenetics patients. Comparing the indirect efficacy results, cilta-cel performed better in terms of ORR, CR rate, DOR, and PFS. However, because of the amount of data, one must exercise caution while analyzing KarMMa (22).

It is challenging to assess the efficacy of CAR-T products in the absence of a head-to-head comparison or randomized controlled study. Cilta-cel has a longer manufacturing time of 4–6 weeks, although this is evolving. In patients whose myeloma is not sufficiently cytoresistant, cilta-cel may potentially raise the risk of atypical neurotoxicity. Because there is less chance of atypical neurotoxicity with ide-cel, patients with neurological diseases at baseline are better candidates. Individuals with serious baseline neurological problems may be more suitable for a BsAb. Patients with bulky disease that is difficult to cytoreduce due to a lack of options for bridging to CAR-T therapy may be at increased risk of progressing to any CAR-T therapy, especially cilta-cel (20). Relapsed MM cells may respond to BCMA-directed therapies or another CAR-T product due to BCMA expression and loss of CAR-T cell persistence (23).

## 3 Early and late toxicity

CAR-T cell therapy has completely changed the course of cancer diseases, especially those of hematology, such as MM. KarMMa and CARTITUDE-4 studies suggest that patients with RRMM can have prolonged maintenance-free remissions after CAR-T cell therapy, albeit with a continued risk of disease progression over time. One of the biggest challenges of CAR-T cell therapy is the management of short- and long-term toxicities. Management of short-term toxicities is already being done successfully. Long-term results are expected. We outline a few of these toxicities, especially short-term toxicities (24).

### 3.1 CRS and ICANS

Immune-related toxicities, including CRS and ICANS, must be treated immediately. The cytokine signaling cascade controls the frequency and severity of CRS. The main cytokine in CRS, IL-6, is responsible for CRS, while the mechanisms for ICANS are not fully elucidated. It is observed in pivotal trials and real-world studies that the high-grade CRS rates were low, and just one grade 5 trial reported CRS incidents utilizing both ide-cel and cilta-cel (4, 15). Although CAR-T cell products vary, CRS usually appears during the first week after cell infusion. To unify grading across all institutions for clinical research and the grading of toxicities in patients receiving CAR-T cell products, the American Society for Blood and Marrow Transplantation (ASBMT) has produced a consensus grading system for CRS (Table 3). The neurological side effects of CAR-T cell treatment are referred to as ICANS, according to the ASBMT recommendations (Table 4) (25). Neurological toxicities generally develop within 4–6 days, with a median duration of 1–14 days (18). Close observation is recommended for CRS and ICANS to enable early detection and intervention that may help stop the onset of adverse events (17, 18). In the US, there is an FDA mandate to be within 2 h of the authorized treatment center to monitor and promptly treat CRS and ICANS (24, 26).

**Table 3:** Grading and management of CRS.

	Grade 1	Grade 2	Grade 3	Grade 4
Fever*	≥38°C	≥38°C	≥38°C	≥38°C
With either (Hypotension)^#^	None	Not requiring vasopressor	Requiring vasopressor	Requiring vasopressor
And/or (Hypoxia)^#^	None	Requiring low-flow nasal cannula	Requiring high-flow nasal cannula, face mask, facemask non-rebreather mask, or venturi mask	Requiring positive pressure (e.g.,> CPAP. BIPAP, intubation, and mechanic ventilation)
Management	Supportive care including analgesics and antipyretics, Consider tocilizumab for persistent (lasting >3 days) and refractory fever	IV fluid bolus Consider tocilizumab 8 mg/kg +/−dexamethasone 10 mg IV 6 h If persistent hypotension, consider transfer to ICU	Consider transfer to ICU Administer Tocilizumab and add steroids if unresponsive within 24 h as Grade 2 If unresponsive CRS add Anakinra	Transfer to ICU Administer tocilizumab High-dose methylprednisolone 1 g/day IV If unresponsive CRS add anakinra If unresponsive, consider alternative agents such as anti-TNF, and other agents as appropriate.

^*^In patients who have CRS then received tocilizumab or steroids, fever is no longer required to grade subsequent CRS severity.

^#^Hypotension and hypoxia should not be attributable to any other cause.

**Table 4:** Grading and management of ICANS.

	Grade 1	Grade 2	Grade 3	Grade 4
ICE score	7–9	3–6	0–2	0 (unarousable)
Depressed level of consciousness	Awakens spontaneously	Awakens to voice	Awakens only to tactile stimulus	Patient is unarousable or requires vigorous or repetitive tactile stimuli to arouse
Seizure	N/A	N/A	Clinical seizure that resolves rapidly; or non-convulsive seizures on EEG, resolves with intervention	Life-threatening prolonged seizure (>5 min); or Repetitive seizures without return to baseline in between
Motor findings	N/A	N/A	N/A	Deep focal motor weakness
Increased Intracranial pressure/cerebral edema	N/A	N/A	Focal/local edema on neuroimaging	Diffuse cerebral edema on imaging
Management with no concurrent CRS	Offer supportive care; aspiration management and IV hydration	supportive care as grade 1 add low-dose dexamethasone for high-risk products or patients*	Transfer to ICU Supportive care and dexamethasone treatment as grade 2	Transfer to ICU consider mechanical ventilation for airway protection high-dose corticosteroids** treat convulsive status epilepticus as per institutional guidelines
Management with concurrent CRS	Consider tocilizumab 8 mg/kg # Consider lorazepam or haloperidol for agitated patients.	Tocilizumab 8 mg/kg# If refractory to tocilizumab, add low-dose dexamethasone* Consider transfer to ICU if neurotoxicity is associated with grade ≥ 2 CRS	Tocilizumab 8 mg/kg# If refractory to tocilizumab, add low-dose dexamethasone* Transfer to ICU	Tocilizumab 8 mg/kg# If refractory to tocilizumab, add high-dose dexamethasone** Transfer to ICU

^*^Low-dose dexamethasone 10 mg IV (or equivalent) can be repeated every 6 h if symptoms worsen. Rapidly taper steroids as clinically appropriate once symptoms improve to grade 1.

^**^High-dose methylprednisolone 1,000 mg/day IV one to two times for 3 days followed by a rapid taper after improvement to grade 1.

^#^Tocilizumab should not exceed 800 mg/dose; it can be repeated every 8 h as needed. Limit to a maximum of three doses in 24 h; a maximum total of four doses.

Weak models have been validated to predict CRS and ICANS. Numerous research investigations have documented correlations between the severity of CRS and ICANS and clinical, biochemical, and product factors (27). CRS and ICANS have been managed using a variety of techniques. Corticosteroids and the IL-1 receptor antagonist anakinra are used in the treatment of CRS and ICANS (28, 29).

### 3.2 IEC-HS

Other serious adverse effects include immune effector cell-associated hemophagocytic lymphohistiocytosis-like syndrome (IEC-HS), hypogammaglobulinemia, and cytopenias (recently dubbed immune effector cell-associated hematotoxicity, ICAHT) (30).

There may be a significant overlap between the CRS and IEC-HS diagnostic criteria. In 22% of patients receiving BCMA-specific CAR-T treatment, an IEC-HS-like hyperinflammatory response was noted. The two main treatments are anakinra and high-dose steroids. Ensuring the safety of CAR-T cell therapy in patients treated earlier in their condition requires early identification, prompt interposition, and treatment strategy formulation (18, 31).

### 3.3 Immune reconstitution, risk of infection with cytopenia, and hypogammaglobulinemia

Cytopenia, including severe cytopenia, is common after BCMA CAR-T. In Hansen et al. (18) research, of patients receiving ide-cel, 60% had anemia, 38% had neutropenia, and 59% had thrombocytopenia; after 1 month, almost 65% had grade 3 cytopenia. Supportive care is critically important and stem cell boosts have been used in some cases to recover counts (32).

CAR-T therapy for BCMA causes plasma cell aplasia, which can lead to severe hypogammaglobulinemia and an augmented risk of infection (33). The risk of infection is greatest in the first few months (34).

On the other hand, hypogammaglobulinemia can last for a very long time. There is limited evidence or consensus about intravenous immunoglobulin (IVIG) therapy for post-CAR-T prophylaxis. Preventive IVIG therapy is recommended by numerous experts when the IgG level drops below 400 mg/dL, even in the absence of severe or recurrent infections in the first few months after BCMA treatment with CAR-T (18).

Patients are on PJP and antiviral prophylaxis till CD4 counts are >200; (add the measurement value – refer to the article) and are offered immunizations post-CAR-T (33).

### 3.4 There are long-term toxicities, including the risk of second primary malignancies

In November 2023, the FDA announced a risk of T cell malignancies in patients who received BCMA CAR for myeloma. Elsallab et al. (35) published incidences of malignancy in CD19 and BCMA therapies. There were only 17 cases of T cell non-Hodgkin lymphoma (3.2% of all second primary malignancies) and only 2 of these were anaplastic large cell lymphoma in patients who received cilta-cel. The rest were in CD19 CAR-T recipients. In this study, the most common second malignancy was myelodysplasia (38% of all second malignancies and 1.7% of adverse events), followed by acute myeloid leukemia (20% of second malignancies and 0.9% adverse events) and skin cancers (10% of second malignancies and 0.4% of adverse events). Longer follow-up is needed to determine the incidence of second primary malignancy and non-relapse mortality due to these cancers post-CAR-T.

## 4 Future directions

### 4.1 Higher efficacy, mechanism of relapse

The prognosis of patients who have not responded to CAR-T cell therapy is poor and still represents an unmet medical need. However, relapse remains the biggest obstacle that needs to be addressed. Recent discoveries have uncovered the mechanisms underlying relapse after CAR-T cell therapy.

Two interactions that are closely related to resistance mechanisms include anti-BCMA CAR-T cells, tumor cells, and the complex tumor microenvironment (TME): antigen escape and CAR-T cell depletion/exhaustion. A few potential approaches to address resistance to CAR-T cell therapy include the use of dual-targeted and armored CAR-T cells, genetic modification to block signals associated with intracellular depletion, small-molecule drugs, bridging therapy, and the selection of T cells obtained at the early stages of the disease for CAR-T cell production (6). For high-risk newly diagnosed patients with multiple myeloma (NDMM), additional treatment options are required since their prognosis is often poor even with standard first-line therapy. CAR-T cell therapy may provide them with a potential cure and a first-line therapeutic option. A report on current multicenter research (NCT04935580), including BCMA/CD19 dual-targeted FasT CAR-T cells in patients with NDMM, was presented at ASH 2022. It reports that 100% of the 13 high-risk NDMM patients treated with BCMA/CD19 dual-targeted rapid CAR-T cells experienced a clinical response, and 69% achieved a sCR, following a median follow-up of 5.3 months. These results demonstrated the safety and efficacy of CAR-T cell therapy in eliciting notable responses in high-risk MM patients treated with prior therapies, making it more widely available to these patients (6).

### 4.2 Novel targets

There are novel targets that are being evaluated for future structures, such as GPRC5D and FcRH5. Talquetamab is an approved GPRC5D targeting bispecific therapy, which has been shown to have good outcomes (36).

### 4.3 Improved manufacturing platform

The 4–6 week manufacture time lends itself to risk of decompensation and death of patients while waiting for manufacture. There is an increasing awareness that decreasing the “brain to vein” time is important for better outcomes. Studies evaluating shorter manufacturing platforms are being conducted. Allogeneic products, which provide “off the shelf” CAR-T, completely bypassing the manufacturing process, are an attractive alternative to autologous CAR-T trials evaluating allogeneic BCMA CAR-T include (37, 38).

### 4.4 Access globally including in Turkey

There are many barriers to CAR-T within the USA. The availability of BCMA CAR-T globally varies, and presently no CAR-T is available for commercial/standard use in Turkey. This is an important perspective since these are therapies with high efficacy (39, 40).

## 5 Conclusion

In conclusion, CAR-T therapies are promising for patients with advanced hematological cancers with limited treatment options. It is important to improve patient access to these therapies. In recent years, anti-BCMA CAR-T cell therapy has achieved remarkable results in R/R MM, and its side effects have been generally manageable. However, relapses still occur after anti-BCMA CAR-T cell therapy, and the high production costs and longer production cycles of autologous CAR-T cell products are some of the major challenges that remain to be addressed, such as limiting their accessibility. Therapeutic strategies currently under investigation include the identification of novel therapeutic targets, optimizing CAR constructs and genetic modification methods, implementing dual-target CAR-T cell therapy, and combining CAR-T cell therapy with other approaches. However, follow-up anti-myeloma therapy remains an important clinical requirement because of persistent high-risk factors and resistance to CAR-T cell therapy.
